# 3D guided large osteochondral allograft reconstructions for post-traumatic osteoarticular bone loss of the knee: a technical note

**DOI:** 10.1007/s00068-026-03133-4

**Published:** 2026-03-03

**Authors:** Nick Assink, Hugo C. van der Veen, Job N. Doornberg, Peter A. J. Pijpker, Frank F. A. IJpma

**Affiliations:** 1 3D Lab, University of Groningen, University Medical Center Groningen, HPC BB75, Hanzeplein 1, Groningen, 9713 GZ The Netherlands; 2 Department of Trauma Surgery, University of Groningen, University Medical Center, Groningen, The Netherlands; 3 Department of Orthopaedics, University of Groningen, University Medical Center, Groningen, The Netherlands; 4 Department of Orthopedic Trauma, Flinders Medical Centre, Flinders University, Adelaide, Australia

**Keywords:** Three dimensional, 3D, Virtual surgical planning, Allograft, Tibial plateau fracture, Distal femur, Osteochondral, Reconstruction, Malunion

## Abstract

**Purpose:**

Post-traumatic osteoarticular bone loss of the knee remains challenging to treat. Allograft reconstruction can yield favourable outcomes, and 3D virtual surgical planning may further improve results by enabling precise templating, optimal graft selection, and accurate fixation. This study aimed to introduce and evaluate 3D-assisted planning for knee allograft reconstruction.

**Methods:**

In two patients with severe post-traumatic knee osteoarticular bone loss—one in the medial distal femur and one in the proximal tibial plateau—3D-assisted resection and allograft reconstruction were performed. An allograft was matched to the affected condyle, after which cutting planes were defined and patient-specific guides were designed. Postoperative CT-scans were obtained to assess accuracy, and patient-reported outcomes were evaluated with the Lower Extremity Functional scale(LEFS) questionnaire.

**Results:**

Both surgical procedures went uneventful. In the lateral plateau case, 3D analysis of the postoperative CT scan showed a maximum translational deviation from the plan of 2.2 mm and a maximum rotational deviation of 3.2°. In the medial distal femur case, postoperative 3D assessment revealed a maximum translational deviation of 2.5 mm and a maximum rotational error of 3.5°. Functional outcomes (> 1 year postop) were favourable, with LEFS scores improving from 29/100 to 78/100 in the lateral plateau case and from 22/100 to 81/100 in the medial distal femur case.

**Conclusion:**

In conclusion, this technical note demonstrates the feasibility and accuracy of 3D-assisted planning for knee allograft reconstruction. The approach enables precise allograft placement and anatomical restoration.

## Introduction

Post-traumatic osteoarticular bone loss of the knee joint represents one of the most difficult challenges in orthopaedic trauma care. The initial injury is often characterized by complex intra-articular fracture displacement, comminution, cartilage defects, and associated soft tissue injuries; all factors that impact functional outcome [1, 2]. As a result, initial surgical treatment may lead to suboptimal reduction in up to 30% of fractures, or may not be constructible at all in rare cases [3]. Malunion around the knee, can have a significant impact, as deviations in leg alignment may lead to pain, instability, progressive osteoarthritis and, ultimately, early conversion to total knee arthroplasty (TKA) [4, 5]. Corrective osteotomy, either extra- or intra articular, is one of the treatment options for restoring alignment and function [6]. However, when the articular surface is severely damaged, or when dealing with significant osteoarticular bone loss, osteotomy alone may not be sufficient. In such cases, joint replacement becomes the only viable solution. Yet, in younger patients, prostheses come with concerns about implant longevity, activity restrictions, and the high likelihood of revision surgery [7].

An alternative treatment option is the use of a large osteochondral allograft – harvested from a human donor - to replace the damaged femoral or tibial condyle in this young population. Satisfactory long-term outcomes have been reported in young, active patients when reconstructing both the tibia and femur using osteochondral allografts to treat large articular cartilage defects [8, 9]. However, in 15–20% of patients, the allograft fails within 10 years, with failure rates increasing to up to 54% at 20 years, highlighting the need for further improvement as well as follow-up studies [8, 9]. In these preliminary series, these allografts were often harvested freehand from the donor bone and subsequently transplanted freehand into the recipient site. This technique may be prone to coronal and sagittal plane malalignment, size mismatches, and inaccuracies. Successful outcomes depend on anatomical and dimensional matching of the articular surface, restoration of joint stability through host–donor soft-tissue repair, and accurate joint alignment; all of which have been linked to reduced degenerative changes in the articular surface of osteoarticular allografts [10]. 3D virtual surgical planning may further improve accuracy by enabling precise pre-operative templating, optimal allograft size selection, joint space morphology matching and ultimate positioning with fixation. The creation of patient-specific cutting guides ensures such anatomical restoration of joint line, mechanical axis, and articular congruity [11, 12]. In this study, we explore whether 3D virtual surgical planning may indeed enable more accurate allograft reconstructions, tailored to patient’s specific knee size, shape, and alignment, within the clinical scenario of young patients with severe post-traumatic osteoarticular bone loss of the knee joint.

The aim of this study was to introduce the concept of 3D guided allograft reconstruction of post-traumatic intra-articular bone loss of the knee and assess its feasibility and accuracy. This technical note describes the full approach, encompassing allograft selection, 3D virtual planning, surgical guide design, 3D-assisted surgery, and postoperative assessment.

## Methods

A 3D guided knee reconstruction with a fresh frozen osteochondral allograft was performed in two patients. This technical note outlines the complete workflow in two illustrative cases involving reconstructions of the lateral tibia plateau and the medial femoral condyle.

### Case 1– 3D guided osteochondral allograft reconstruction of the lateral tibial plateau

**Fig. 1:**
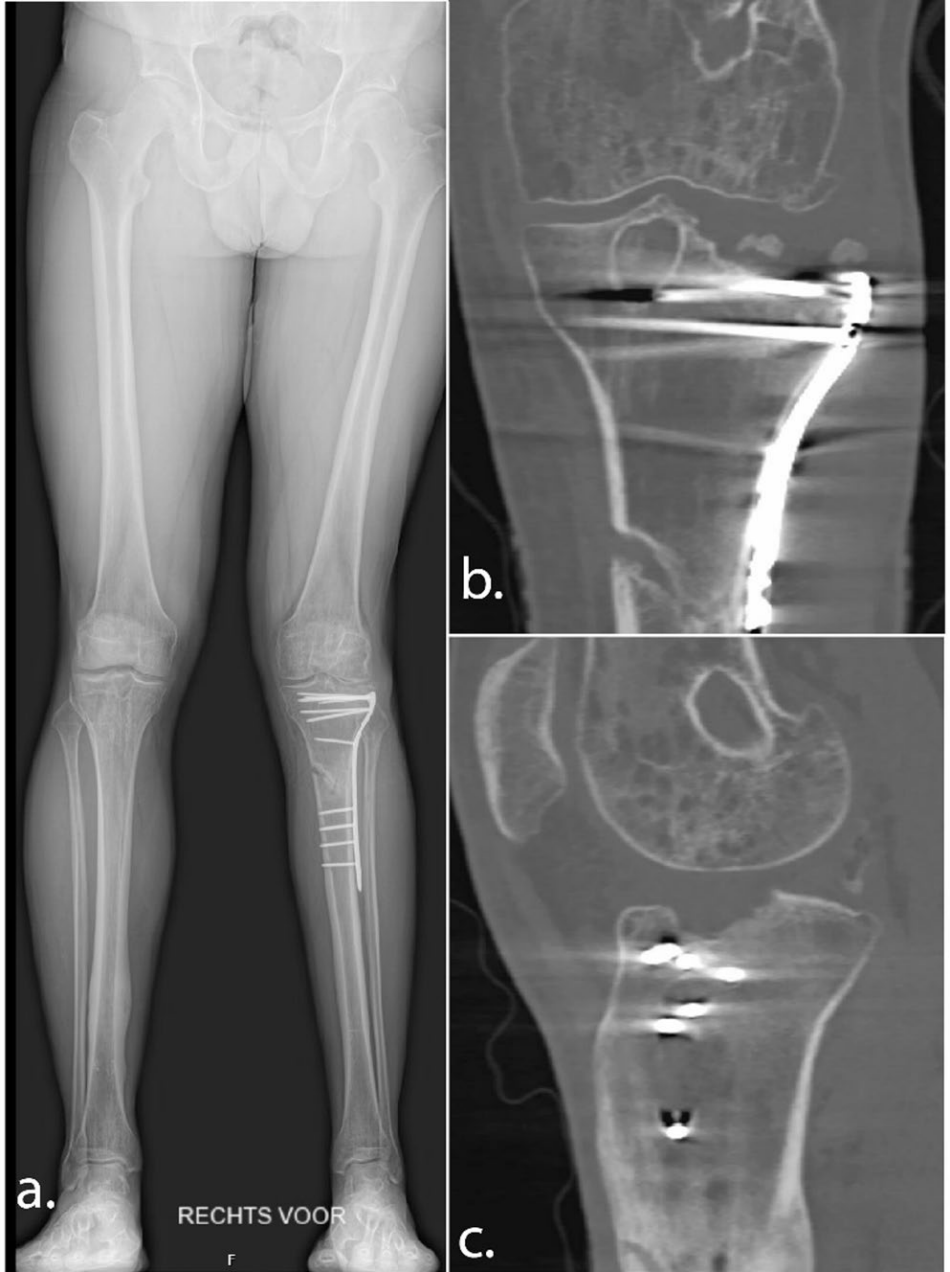
The long leg radiograph (**a**), coronal CT-image (**b**) and sagittal CT-image (**c**) illustrating the severe lateral plateau damage from the first outpatient visit at our clinic from the first case

The first patient was a very active and athletic 40-years-old male, with a medical history of an anterior cruciate ligament injury and successful reconstruction with return to sport, who was referred to our clinic with severe osteoarthritis of the lateral tibial plateau, 8 months following a combined tibial plateau and shaft fracture. His main complaints included severely limited knee function, the ability to walk only 10 min with the support of a stabilizing brace, progressive lateral knee pain, valgus instability and a clinically evident valgus malalignment of the affected leg. At the time of injury, he underwent surgery at a different hospital with open reduction and lateral plate osteosynthesis. During this index procedure, intraoperatively the fracture was found to be extensively comminuted and could not be adequately reconstructed (Fig. 1). Follow-up radiographs and CT scans revealed a severe osteochondral injury with depression and comminution of the lateral tibial plateau. Given that the patient was young and athletic, had a complete destruction of the lateral tibial plateau, and did not want a knee arthroplasty in a shared decision-making process, he opted for a large osteochondral allograft of the entire lateral tibial plateau.

### Virtual surgical planning

**Fig. 2:**
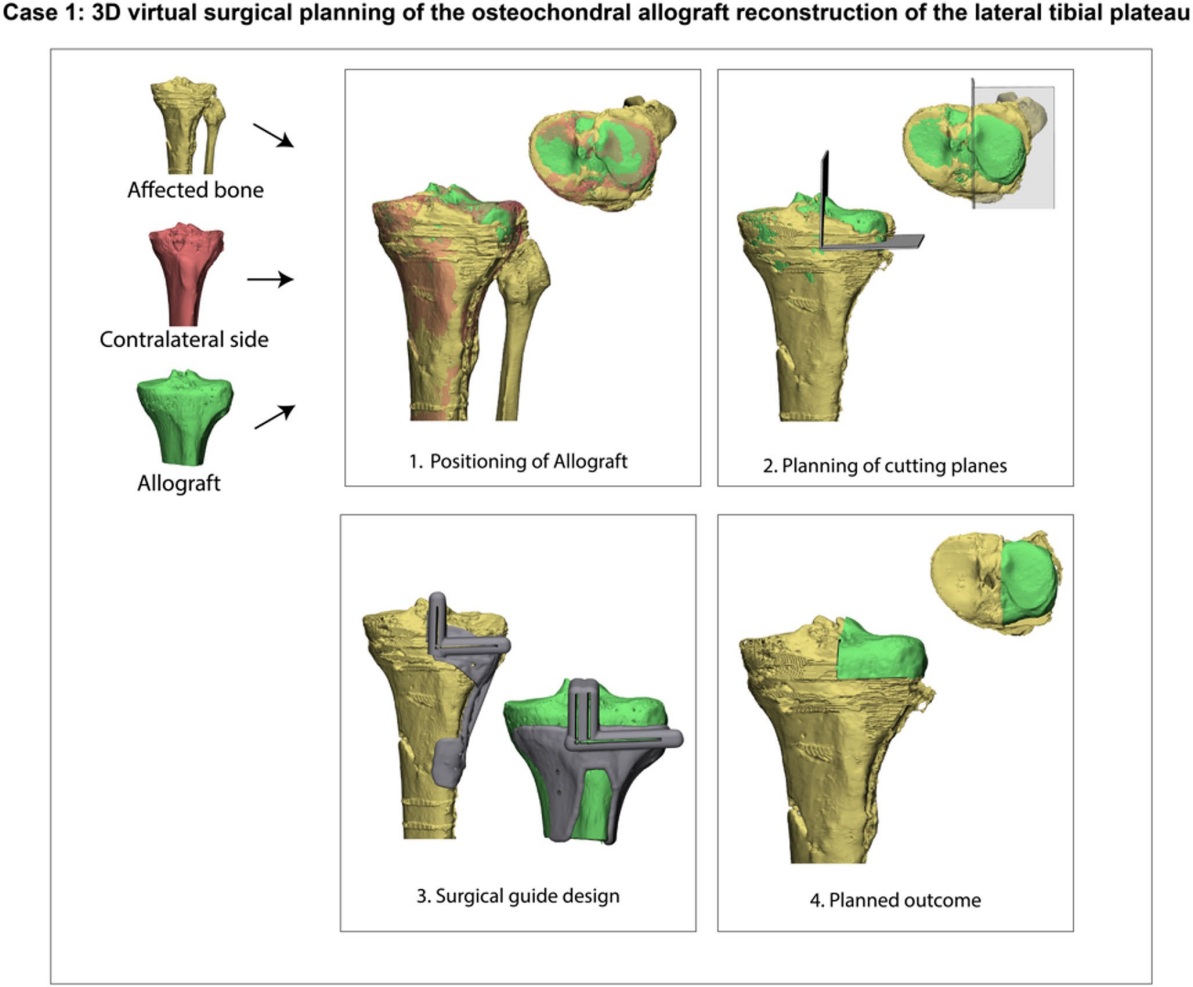
Virtual surgical planning for a lateral tibial plateau reconstruction. The healthy contralateral bone was first mirrored and registered to the affected side to quantify the size of the defect. A three-dimensional model of the allograft was then aligned to the mirrored contralateral condyle to re-establish native joint geometry. Osteotomy planes were placed at least 2 mm from the defect margins (based on surgical guide accuracy) with an acute angle (< 90 degrees) to prevent slippage of the allograft and a press-fit interface. Finally, patient-specific cutting guides were designed to translate these virtual cutting planes to the actual surgery to achieve the planned anatomic reconstruction

Following a preoperative bilateral CT-scan, a 3D reconstruction of the affected knee was created using Mimics software (version 25.0, Materialise, Leuven, Belgium). Measurements of the contralateral femoral and tibial condyles were performed and sent to the bone donor bank (ETB-Bislife, Haarlem, the Netherlands), after which a matching donor bone was identified. A CT-scan of the allograft was performed and sent to our Academic institution to verify the match in our 3D Lab (www.3Dlabgroningen.nl). After successful identification and match of the allograft, a virtual surgical plan was created using 3-Matic software (Version 17.0, Materialise, Leuven, Belgium). After virtually fitting the allograft to the defect, cutting planes were placed to ensure optimal alignment with the recipient bone. Furthermore, to prevent slippage of the allograft, the two cutting planes in the anteroposterior view were positioned at an acute angle (< 90 degrees), with the axial plane positioned perpendicular to the loading axis. This to safeguard optimal biomechanical distribution of compressive forces to facilitate ingrowth of the graft. Additionally, two options for the vertical cuts were planned: one preserving and one resecting the meniscal origin. The final choice was made intraoperatively, depending on the condition of the meniscus. To translate the virtual plan into the operating room, patient-specific 3D-printed surgical guides were developed and produced. Initial surgical guide designs were first tested on Thiel-embalmed cadavers (Appendix 1). Based on these tests, the designs were optimized with consideration for the soft tissue constraints associated with the planned surgical approaches. Figure 2 illustrates the virtual surgical planning for the first case. After virtual design of the surgical plan and guides, the digital files of the surgical guides were sent to a specialized 3D printing facility (Oceanz 3D Medical Models, Ede, the Netherlands) and manufactured using selective laser sintering (SLS) 3D printing with PA 12 (polyamide) under an ISO 13485–certified workflow. The guides were then prepared for surgery via routine steam sterilization at 134 °C, while the pre-matched allograft was received sterile-packed.

### Surgery

**Fig. 3:**
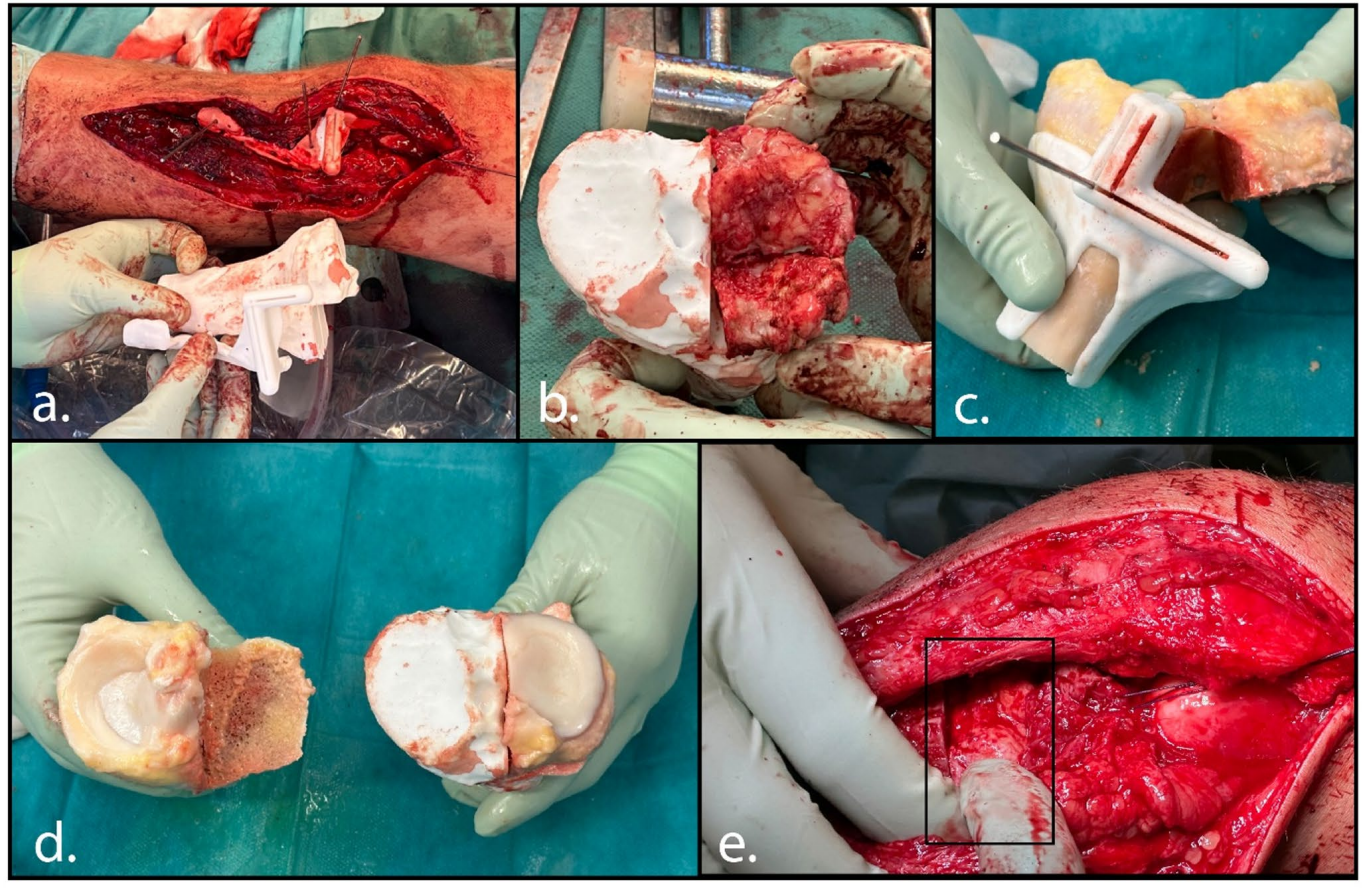
Surgical procedure of case 1. Lateral view of the left knee showing the lateral tibial plateau through an anterolateral approach. First the surgical cutting guides was placed on the native bone to resect the affected lateral tibial plateau (**a**). The resected tibial plateau was checked using a 3D print including the non-resected part (**b**). The corresponding lateral plateau of the allograft was harvested with a surgical guide (**c**). The harvested allograft was checked with a print intended position (**d**). Lateral view on the left knee showing the allograft (black box) positioned in the lateral defect and secured with three headless compression screws (**e**)

An anterolateral approach to the tibial plateau was performed, and the plate from index surgery was removed. A 3D-printed osteotomy guide, with pins fitting the former screw holes for reproducible placement, was then positioned on the lateral tibial plateau and temporarily fixed with K-wires (Fig. 3). An arthrotomy was performed, while the lateral meniscus was elevated and left in situ throughout the procedure. Guided by the 3D-printed osteotomy guide, the severely damaged lateral tibial plateau was resected using an oscillating saw (Stryker Precision Falcon, 1.28 mm thick) and osteotomes. The resected segment measured 22 × 38 × 64 mm. Particular attention was given to ensure that the sagittal osteotomy plane was placed just lateral to the root insertions of the native lateral meniscus. After removal of the plateau, a longitudinal tear in the anterior part of the lateral meniscus was identified and repaired. A 3D-printed osteotomy guide was then applied to the allograft, allowing harvesting of the lateral tibial plateau graft. After the donor allograft was irrigated with pulse lavage to remove intramedullary fat as much as possible, the allograft was implanted and fixed with three headless compression screws, after which the wound was closed. Rehabilitation consisted of 8 weeks of non-weight-bearing mobilization, followed by a gradual increase in weight-bearing: 40 kg for 4 weeks, then 60 kg for 4 weeks, and finally progression to full weight-bearing.

### Case 2–3D guided osteochondral allograft reconstruction of the medial femoral condyle

**Fig. 4:**
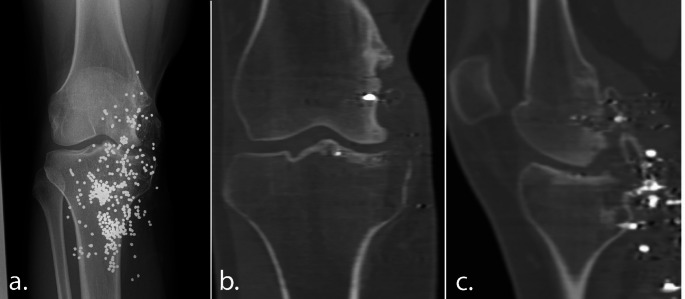
The knee radiograph (**a**), Coronal CT-image (**b**) and sagittal CT-image (**c**) from the second case, illustrating severe medial femoral condyle damage caused by shotgun pellets at the time of the first outpatient visit at our clinic

The second patient was a 16-years-old male with a severe destruction of the medial femoral condyle due to a shooting accident abroad, two years prior. Initial treatment had consisted of washout, debridement, direct MCL repair and closure combined with antibiotic treatment. He was referred to our clinic because of residual symptomatic varus instability and progressive pain. Physical examination of his right knee showed a medial sided scar, with an atrophic medial aspect of his knee with a palpable deformation of the medial femoral condyle. Flexion was slightly decreased (F/E: 120/0/0). There was an evident medial defect varus laxity in flexion with also signs of medial collateral ligament attenuation. ACL and PCL were stable. Figure 4 shows the anteroposterior and anteroposterior knee radiographs and CT-scans at the time this patient presented in our clinic.

### Virtual surgical planning

**Fig. 5:**
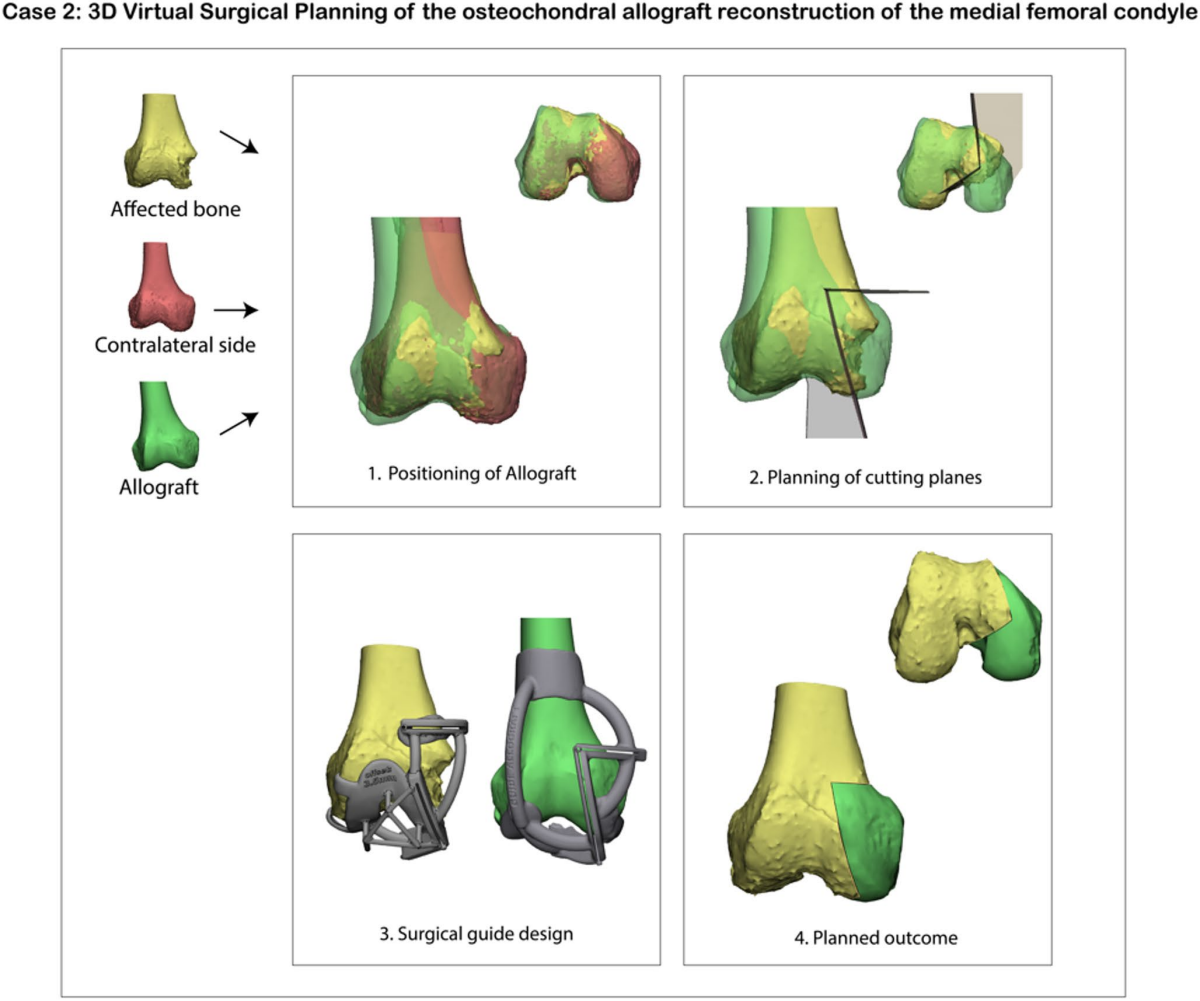
Virtual surgical planning for a distal medial femur condyle reconstruction. The healthy contralateral bone was first mirrored and registered to the affected side to quantify the size of the defect. A three-dimensional model of the allograft was then aligned to the mirrored contralateral condyle to re-establish native joint geometry. Three osteotomy planes were placed at least 2 mm from the defect margins (based on surgical guide accuracy) with an acute angle (< 90 degrees) to prevent slippage of the allograft and a press-fit interface. Finally, patient-specific cutting guides were designed to translate these virtual cutting planes to the actual surgery to achieve the planned anatomic reconstruction

The virtual surgical planning was performed using the exact same method as within the first case. To compensate for size discrepancies in this case, the allograft was only matched to the affected condyle to prevent excessive widening. The guide was designed to fit onto both bony and cartilaginous anatomical surfaces. For seating on cartilaginous areas, an additional offset was incorporated to compensate for cartilage thickness not captured by the CT scan segmentation. Uniquely in this case was that the surgical guide did not contain two, but three cutting planes to optimally resect the affected condyle. Figure 5 shows the virtual surgical planning process in this case.

### Surgery

**Fig. 6:**
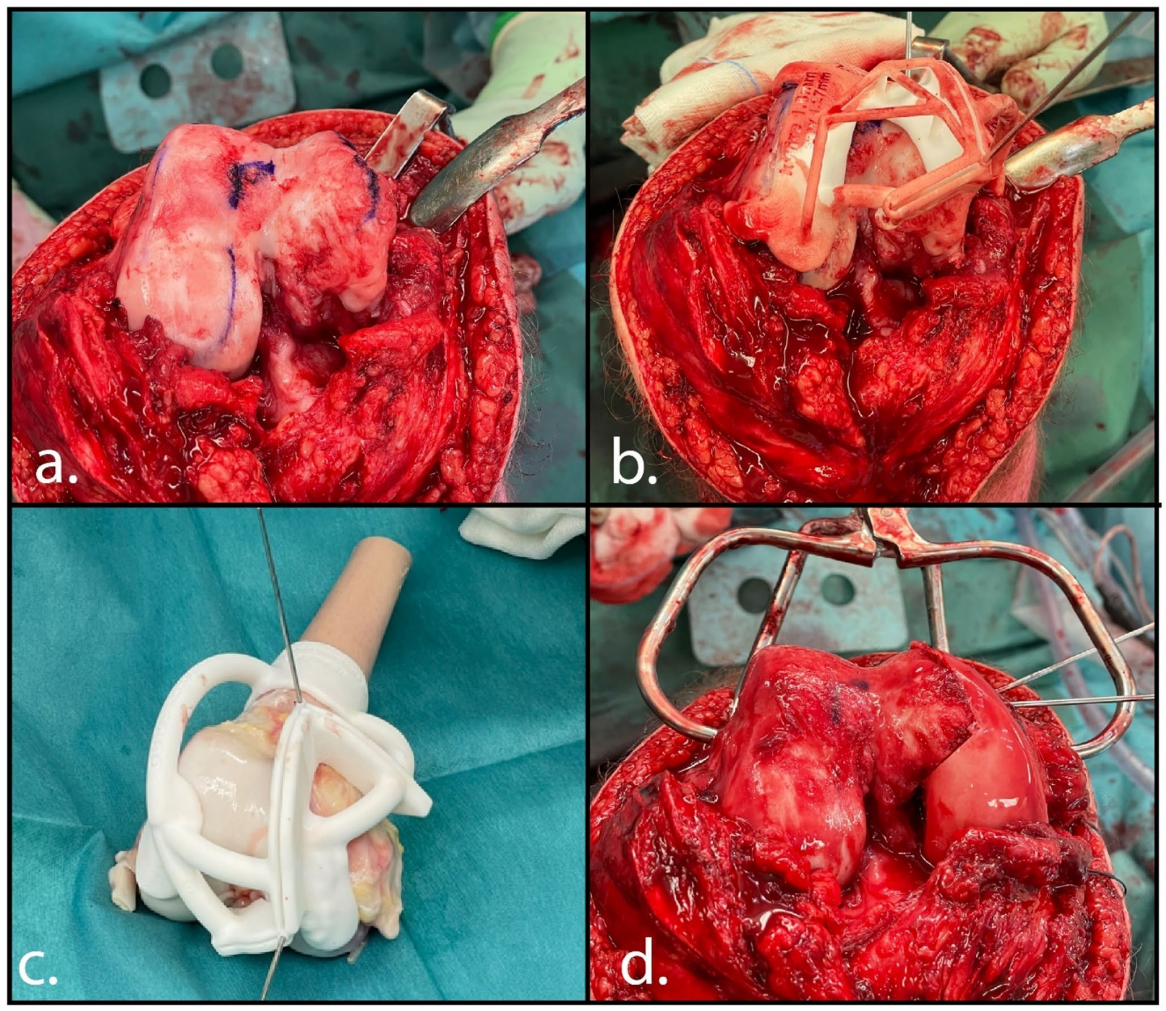
Surgical procedure of case 2. First the femur was exposed and the medial defect was identified (**a**). The surgical cutting guide was placed on the native bone to resect the affected medial femur condyle (**b**). The medial condyle of the allograft was harvested with a surgical guide (**c**). Lastly, the allograft was placed in the medial defect and secured with compression screws and a buttress plate (**d**)

Via midline incision, an anteromedial arthrotomy was performed with lateral reflection of the patella. Both femoral condyles where exposed, and the 3D printed guide was fitted on the condyles with temporary K-wire fixation. With an oscillating saw (Precision, Stryker) and osteotomes the damaged part of the femoral condyle was carefully resected (Fig. 6). Subsequently, the other 3D printed guide was placed and fixed on the allograft and a matching allograft was harvested on table. After thorough irrigation, the allograft was fitted on the recipient condyle and fixed with compression screws and a buttress plate. After closure of the capsule, an achilles tendon allograft was used for reconstruction of the medial collateral ligament. Proximal the tendongraft was firmly sutured to the MCL stump on the allograft, distal fixation was performed by an interference screw. Rehabilitation consisted of an extension splint for 2 weeks followed by a hinged knee brace for 3 months. Weightbearing was not allowed for the first 8 weeks and gradually increased to full weightbearing after 6 months.

### Postoperative outcome

Following surgery, a postoperative CT scan was performed according to standard of care. 3D reconstructions of the postoperative bone were generated, and segmentation was carried out using the same method as in the preoperative workflow. The virtually planned allograft position was then aligned with the postoperative position. Preoperative, planned, and postoperative bone models were exported from the 3-Matic software. Translational errors relative to the planned allograft position were measured in the anterior–posterior, left–right, and cranial–caudal directions (in millimetres). Rotational errors (in degrees) were assessed in the coronal, sagittal, and axial planes. Additionally, functional outcome was assessed both pre- as postoperatively using the Lower Extremity Functional Scale (LEFS) questionnaire [13]. The LEFS score is a validated questionnaire reporting on patient’s functional outcomes with a score of 0 indicating poor knee function, and 100 of perfect knee function.

## Results

### Case 1– 3D guided osteochondral allograft reconstruction of the lateral tibial plateau

**Fig. 7:**
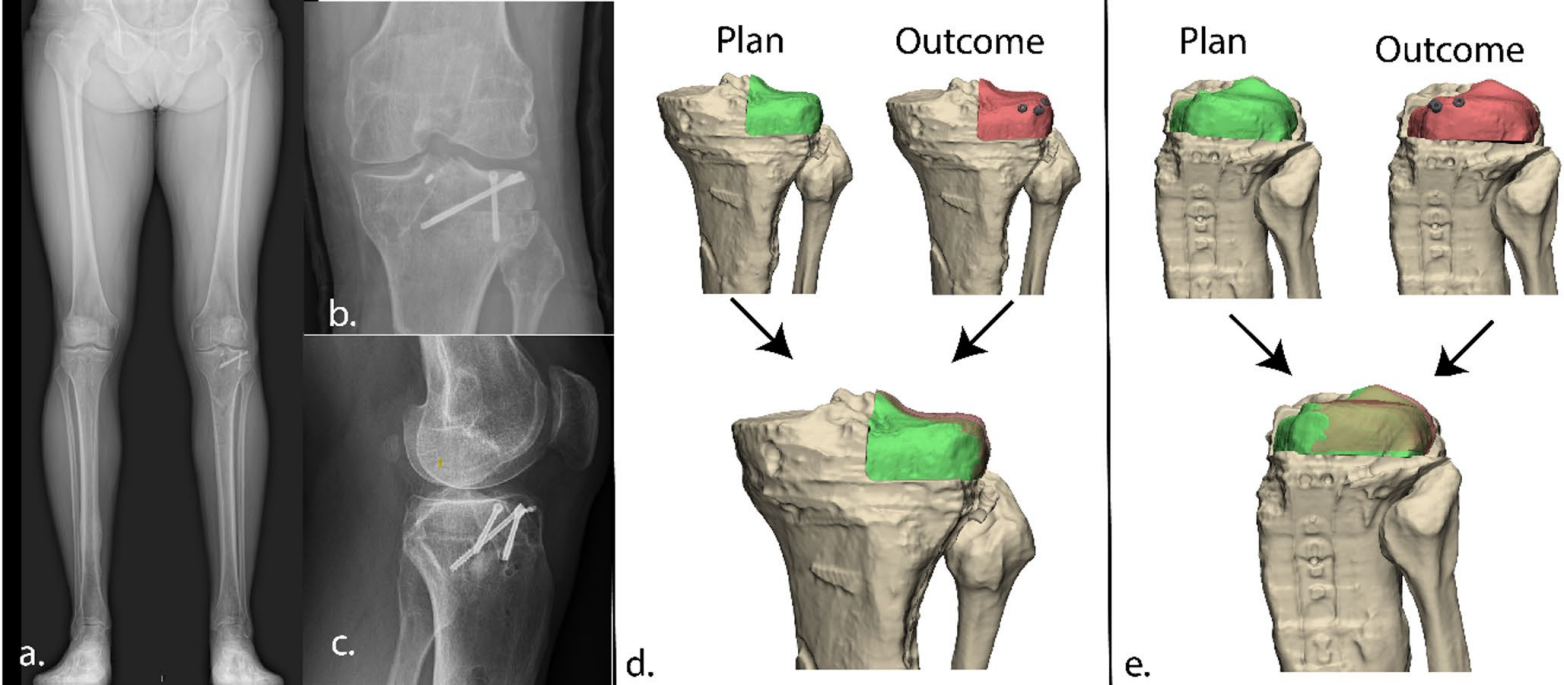
Postoperative outcome. Displacement was analyzed along three axes: anterior-posterior (x-axis), medial-lateral (y-axis), and cranial-caudal (z-axis). The analysis revealed a devation between planned and position, with a posterior displacement of 1.0 mm, a lateral displacement of 0.2 mm, and a cranial displacement of 2.2 mm. Rotational error analysis showed deviation between the planned and achieved allograft position of 3.2° in the sagittal plane, 2.5° in the coronal plane, and 0.5° in the axial plane, indicating accurate placement of the allograft

The surgical procedure went uneventful. A quantitative 3D assessment of the postoperative CT scan was conducted to evaluate the translational and rotational error relative to the planned graft position. As compared to the planned position, a posterior displacement of 1.0 mm (x-axis), a lateral displacement of 0.2 mm (y-axis), and a cranial displacement of 2.2 mm (z-axis) was found. In terms of rotation, a deviation from the planned orientation of the graft of 3.2° in the sagittal plane, 2.5° in the coronal plane, and 0.5° in the axial plan was found (Fig. 7).

**Fig. 8:**
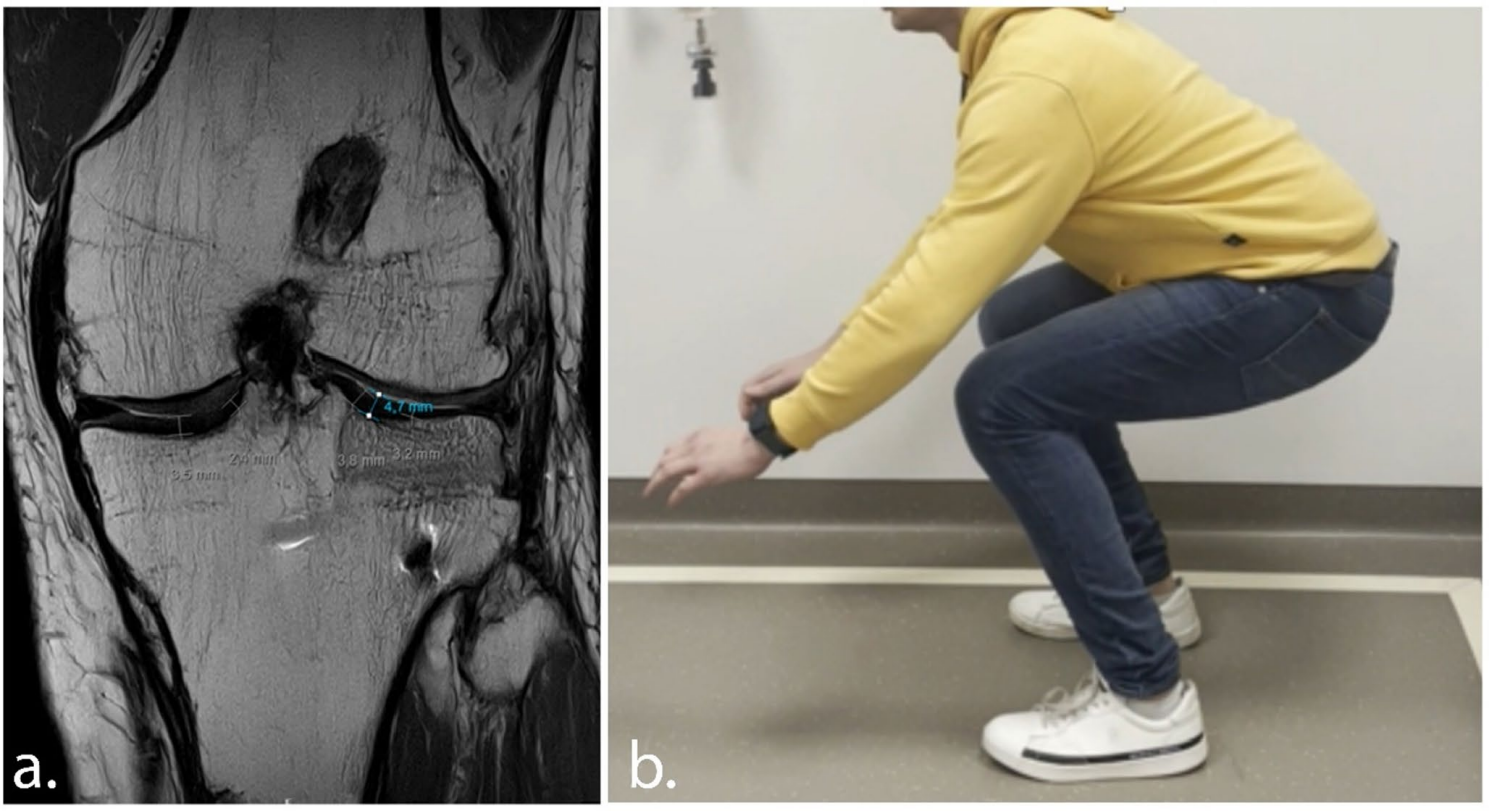
At one-year follow-up, the patient showed favourable clinical recovery, with coronal proton density–weighted turbo spin echo (PD-TSE) MRI of the lateral tibial plateau allograft demonstrating good integration and preservation of cartilage (**a**) and good squatting ability (**b**)

Clinically, the patient was pain-free and satisfied at the 3-month follow-up, with full recovery of knee function observed at one-year follow-up and a reported LEFS score of 78/100 (before reconstruction 29/100). The patient engages in sports twice a week, including fitness training with leg press, squats, and other leg exercises. Additionally, he cycles twice weekly (80 km) and walks 6 km four times per week, but no running. Follow-up radiographs demonstrate successful integration of the allograft with the native bone and neutral knee alignment (Fig. 7). At one-year follow-up, an MRI-scan demonstrated successful integration of the allograft, with maintained height, and cartilage thickness of about 4.5 mm, comparable to that of the healthy medial plateau (Fig. 8). Patient will remain in follow-up care at 2, 5 and 10 years.

### Case 2–3D guided osteochondral allograft reconstruction of the medial femoral condyle

**Fig. 9:**
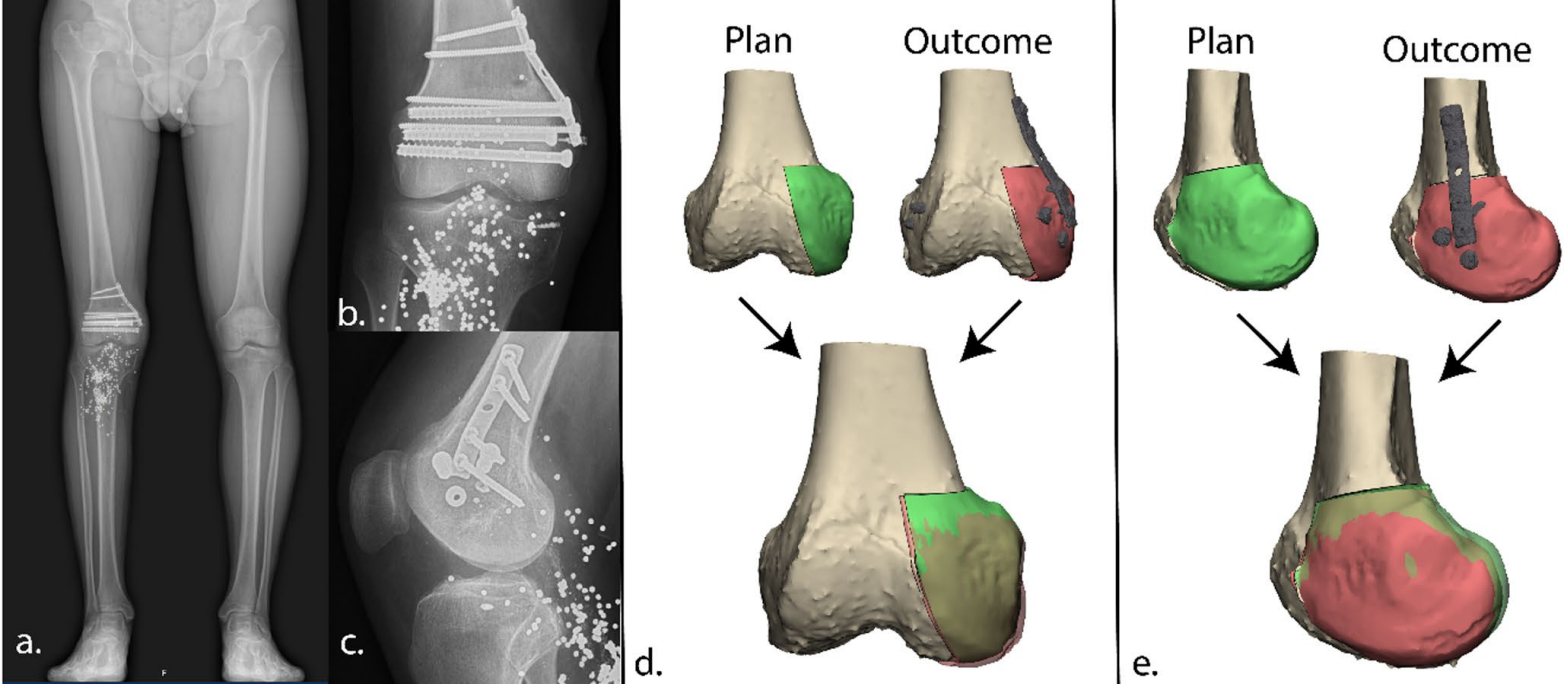
Postoperative outcome. Displacement was analyzed along three axes: anterior-posterior (x-axis), medial-lateral (y-axis), and cranial-caudal (z-axis). The analysis The analysis revealed a devation between planned and position, with a posterior displacement of 2.5 mm, a lateral displacement of 2.0 mm, and a cranial displacement of 1.0 mm. Rotational error analysis showed deviations of 3.5° in the sagittal plane, 0.9° in the coronal plane, and 3.0° in the axial plane

**Fig. 10:**
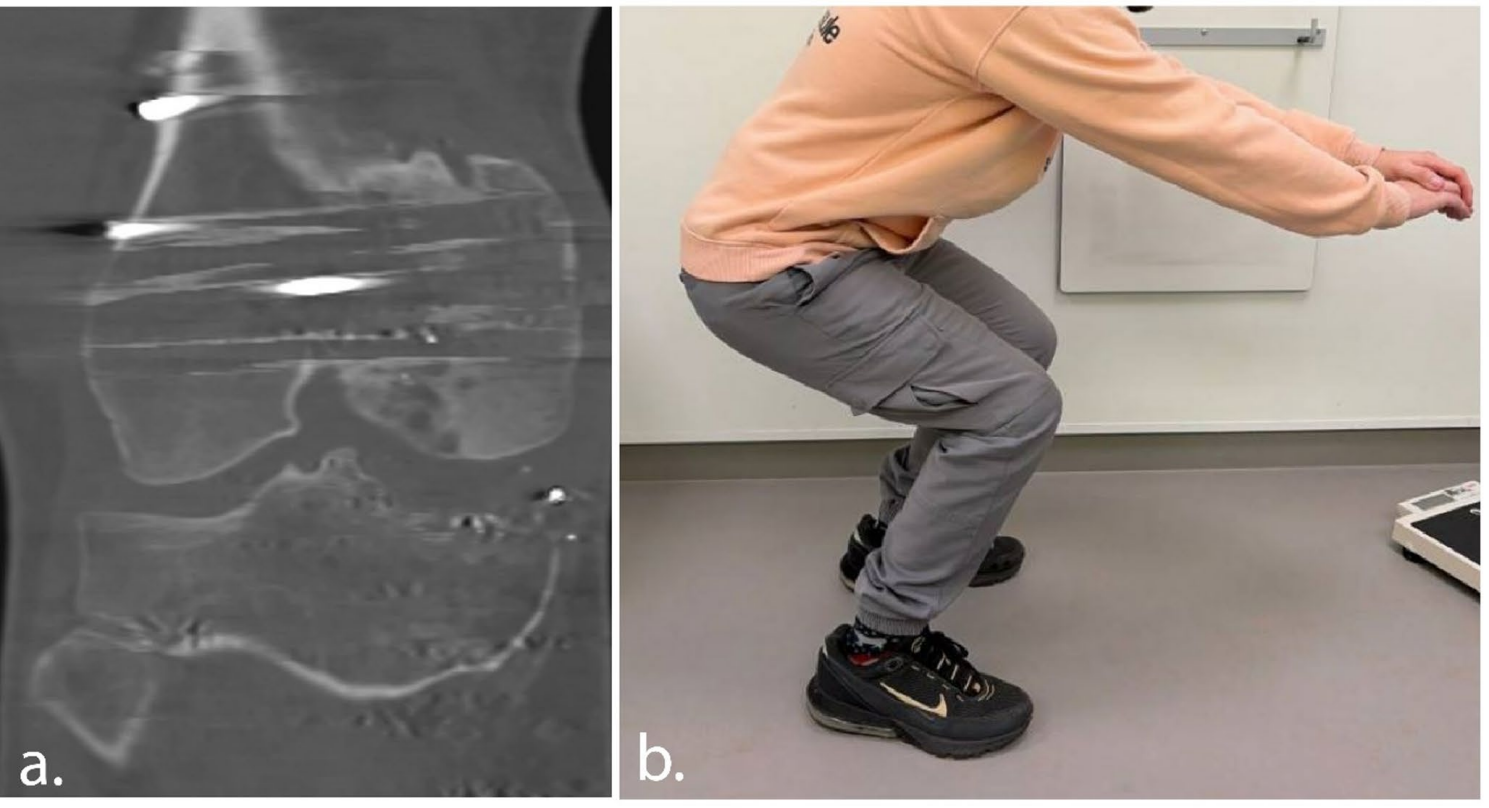
At 15 months follow-up, the patient showed favourable clinical recovery, with the CT-scan of the medial allograft demonstrating good integration (**a**). Due to recurrent medial valgus instability a revision MCL reconstruction was performed. After the final follow-up at three years good functional outcome and squatting ability was observed (**b**)

The surgical procedure went uneventful. Postoperative 3D assessment showed a deviation between the planned and achieved position, with a translational error of 2.5 mm posterior (x-axis), 2.0 mm lateral (y-axis), and 1.0 mm cranial (z-axis). The 3D rotational error was 3.5° in the sagittal plane, 0.8° in the coronal plane, and 3.0° in the axial plane (Fig. 9). Clinical recovery was uneventful and after 3 months more stability was experienced in daily living. Due to recurrent medial valgus instability a revision MCL reconstruction was performed 20 months after the initial procedure; which was probably due to the poor proximal graft fixation (donor-on-donor soft tissue). At final follow-up, three years after osteochondral allograft placement, and one year after MCL revision surgery, patient was active mobile and valgus instability resolved. Patient reported good outcome with 81/100 LEFS score (before initial surgery 22/100). CT scan 15 months after the index procedure revealed bone ingrowth (Fig. 10).

## Discussion

Knee reconstruction using a (fresh-frozen) large osteochondral allograft may be a viable treatment option with favourable functional short-term outcomes, particularly in younger patients for whom arthroplasty is not deemed appropriate. Proper allograft sizing and precise knee alignment are considered critical, as successful outcomes partly rely on modifiable factors such as accurate anatomical and dimensional matching of the articular surface, restoration of joint stability through host–donor soft-tissue repair, and correct joint alignment—all factors associated with reduced degenerative changes in osteoarticular allografts [10]. This technical note introduces a 3D-assisted approach to allograft reconstruction for knee joint injuries, demonstrating not only its feasibility, but also its ability to accurately restore anatomy and the patient’s native morphology.

As one of the body’s primary weight-bearing joints, the knee endures thousands of steps daily, resulting in over one million load-bearing contacts each year. Even minor misalignment of an osteochondral allograft may therefore contribute to early graft failure. Despite the known association between knee malalignment and increased risk of osteoarthritis, placement accuracy is rarely reported in studies on conventional allograft techniques [14–16]. Reported failure rates remain considerable: 9–13% at 5 years, 20–24% at 10 years, and 33–54% at 20 years. Improving placement accuracy may offer a solution toward better graft survivorship. Koulalis et al. demonstrated that navigation significantly improves angle and depth matching during graft harvest and placement compared to the conventional freehand technique, using a customized reference tracker fixed to the donor and recipient [17]. While the use of 3D-printed surgical guides appears to allow for precise resection and placement across various anatomical sites, most studies do not quantify accuracy post-operatively [18–20]. 3D surgical guides have, however, shown accuracies around 2 mm and 3 degrees from the preoperative plan in corrective osteotomy [11, 21]. In our study, we observed comparable accuracy post-operatively, with allograft positioning achieved within 2.5 mm and 3.5° of the intended position. These findings support the feasibility of highly accurate graft placement using a 3D-assisted approach.

The cases presented in this technical note utilized fresh-frozen osteochondral allografts, as matching fresh allografts were not available. At one-year follow-up, radiographs showed graft incorporation and cartilage viability as objectified on advance imaging, as well as functional outcomes appeared regained. Although preservation methods are thought to influence graft viability and clinical outcomes, a recent systematic review reported inconsistent effects of storage media on cartilage health, with 75% of clinical studies showing no clear correlation between preservation method and clinical outcomes [22]. Nonetheless, fresh grafts may be preferable in younger or high-demand patients, where chondrocyte viability is critical, while fresh-frozen grafts remain a practical alternative when fresh tissue is unavailable. However, the limited time window for fresh grafts can restrict optimal sizing and donor matching, which is generally easier with frozen grafts. Although the use of allografts has been described, the long-term viability of the cartilage remains uncertain. Follow-up MRI findings are generally not reported in studies on this topic. Our study adds to the current literature not only by describing the combined use of 3D surgical planning, 3D-printed osteotomy guides, and accurate postoperative 3D evaluations, but also by providing one-year follow-up MRIs demonstrating preservation of the cartilage in the short-term. Longer-term follow-up will be monitored.

A key aspect of this in-house 3D-assisted method was the precise planning and fitting of the allograft to achieve anatomical reconstruction of the knee, thereby offering the patient the best chance at functional recovery. However, during the planning and execution of the procedure, several challenges were encountered. First, with the reconstruction of the tibial plateau the question arose whether or not to replace the meniscus as well. Preoperative assessment of meniscal integrity using MRI was limited due to artifacts from the osteosynthesis material in situ. To account for this uncertainty, we designed two options for the sagittal cutting plane (one including meniscal resection and one preserving the meniscal insertions) allowing intraoperative evaluation of meniscal quality to guide final intra-operative decision-making. In this case, the meniscus appeared to be of good quality and was preserved. Ideally, a preoperative MRI should be available. Not only to assess meniscal integrity but also to evaluate the meniscal insertions, which could further optimize graft orientation and resection planning in case of tibial plateau allografts. Second, the distal femur reconstruction was complicated by extensive damage to the medial collateral ligament resulting from the initial trauma, which also required reconstruction. This was taken into account during surgical planning and the design of the patient-specific guides. Following 3D reconstruction and capsular closure, an achilles tendon allograft was used to reconstruct the medial collateral ligament. This extensive procedure could independently influence patient outcomes, separate from the effects of the osteochondral allograft reconstruction.

This study has some limitations which need to be addressed. First, this technical note involved a limited number of cases, which restricts the generalizability of the findings. Additionally, broader applicability may be influenced by practical factors such as costs, availability of suitably matched donor allografts, and the logistics and processing requirements associated with their use. While our center has access to specialized 3D software and experts for preoperative planning, adoption of this technique may be limited in centers without similar facilities. Also, the follow-up in this technical note was limited to one year following the reconstruction. Since the primary goal of this study was to assess the feasibility and accuracy of the 3D-assisted method, only short-term recovery was evaluated. The high placement accuracy observed in this study represents a clear technical strength, and is expected to contribute to improved load distribution, joint congruency, and graft osseointegration, potentially enhancing graft longevity. However, these clinical benefits cannot be directly demonstrated within the current case-based design and require evaluation in larger, long-term studies. Therefore, future case series with long-term follow-up are needed to evaluate allograft survival, durability, and to confirm the generalizability of this approach.

In conclusion, this technical note demonstrates the feasibility and accuracy of 3D-assisted surgical planning for knee allograft reconstructions. This method yielded accurate allograft placement within 2.5 mm and 3.5° of the intended position, supporting precise anatomical restoration in these illustrative cases.

## Appendix

**Figure:**
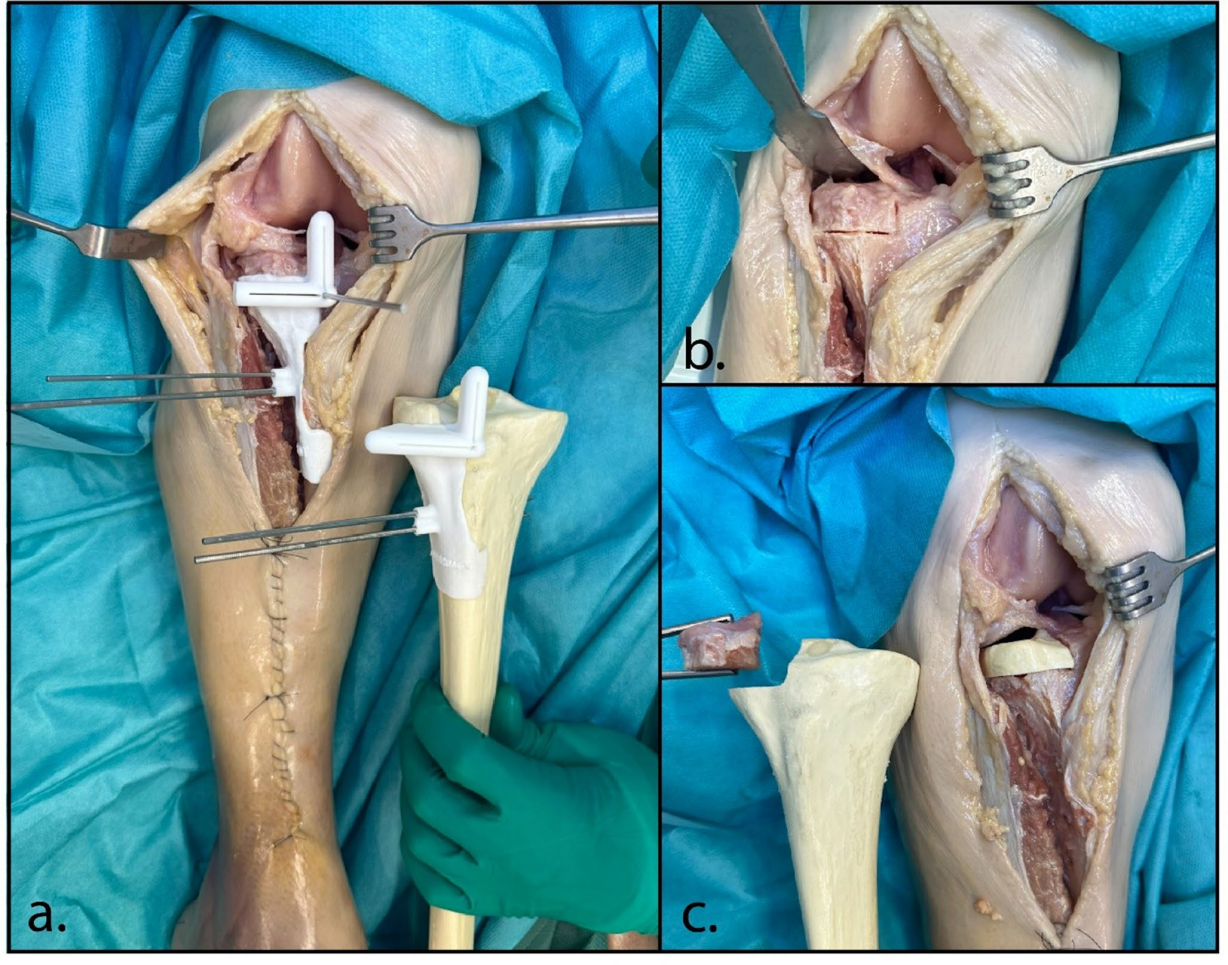
Initial guide designs were first tested on a Thiel-embalmed cadaver. Surgical guides were designed to fit the specimen and a sawbone and incorporate matching cutting planes (**a**). The lateral tibial plateau was then resected using the guide (**b**) and replaced with the harvested lateral plateau of the saw bone (**c**)

## Data Availability

No datasets were generated or analysed during the current study.
